# How do people interpret information about colorectal cancer screening: observations from a think‐aloud study

**DOI:** 10.1111/hex.12117

**Published:** 2013-08-05

**Authors:** Samuel G. Smith, Gemma Vart, Michael S. Wolf, Austin Obichere, Helen J. Baker, Rosalind Raine, Jane Wardle, Christian von Wagner

**Affiliations:** ^1^ Cancer Research UK Health Behaviour Research CentreDepartment of Epidemiology and Public HealthUCLLondonUK; ^2^ Health Literacy and Learning ProgramDivision of General Internal MedicineFeinberg School of Medicine at Northwestern UniversityChicagoILUSA; ^3^ Colorectal UnitDepartment of SurgeryUniversity College London HospitalsLondonUK; ^4^ Community Health and Learning FoundationLeicesterUK; ^5^ Department of Applied Health ResearchUCLLondonUK

**Keywords:** cancer screening, colorectal cancer, fuzzy‐trace theory, literacy, patient information, think aloud

## Abstract

**Background:**

The English NHS Bowel Cancer Screening Programme biennially invites individuals aged 60–74 to participate in screening. The booklet, ‘Bowel Cancer Screening: The Facts' accompanies this invitation. Its primary aim is to inform potential participants about the aims, advantages and disadvantages of colorectal cancer screening.

**Objective:**

To provide detailed commentary on how individuals process the information contained within ‘The Facts’ booklet.

**Design, setting and participants:**

This study comprised of 18 interviews with individuals aged 45–60 and used a ‘think‐aloud’ paradigm in which participants read aloud the booklet. Participant utterances (verbal statements made in response to researcher‐led prompts) were transcribed and analysed using a combination of content and thematic analysis.

**Results:**

A total of 776 coded utterances were analysed (mean = 43.1 per person; range = 8–95). While overall comprehension was satisfactory, several problem areas were identified such as the use of complex unfamiliar terminology and the presentation of numerical information. Specific sections such as colonoscopy risk information evoked negative emotional responses. Participants made several suggestions for ways in which comprehension might be improved.

**Conclusion:**

Public perceptions of the NHS Bowel Cancer Screening Programme information materials indicated that specific aspects of the booklet were difficult to process. These materials may be an appropriate target to improve public understanding of the aims, benefits and disadvantages of colorectal cancer screening. These findings will contribute to a broader NIHR‐funded project that aims to design a supplementary ‘gist‐based’ information leaflet suitable for low literacy populations.

## Introduction

Colorectal cancer is a major cause of mortality in the UK, accounting for 16 000 deaths annually.^1^, ^2^, ^3^ In 2006, the NHS Bowel Cancer Screening Programme (BCSP) was introduced in England for individuals aged 60–69, with plans to extend the age range to 74 by 2014. The programme uses biennial faecal occult blood testing (FOBt), which has been shown to reduce cancer‐specific mortality by 16%.^4^ Participation after the first round of invitations was 54%; however, this varied substantially between socio‐economic groups.^5^ Participation is substantially lower than the breast and cervical programmes that consistently report uptake of 70–80%.^6^, ^7^

The nature of the test and the organization of the programme mean that there is no health‐care professional involvement in the initial stages of screening. The programme therefore relies on communication materials such as ‘Bowel Cancer Screening: The Facts’^8^ to provide information about colorectal cancer and the advantages and disadvantages of screening. This is a 15‐page booklet that is sent out with the initial invitation letter, 2 weeks prior to the screening test kit. It has been translated into 20 different languages and is available in British Sign Language, braille, audio and large print.

A number of determinants of screening participation have been identified.^9^, ^10^, ^11^, ^12^ Less is known about the role of factors that underpin the ability to evaluate and interpret cancer screening information. For example, individuals with low health literacy have been shown to process ‘The Facts’ booklet more slowly and be less interested in learning more about the programme.^13^ Concern has been raised more generally about the length and complexity of the information materials.^14^

Existing research findings are particularly problematic because they suggest that the information materials heavily rely on numerical information and complex terminology that may not be familiar to the public. In turn, this may impede engagement with the aims, benefits and disadvantages of screening. Medical decision‐making theory^15^ and conceptual frameworks^16^ suggest that the increasing tendency to provide more information to the public and patients^17^ may impair the ability to adequately process important information. It is argued that instead of the literal facts supplied by so‐called ‘verbatim’ information, there is a preference for information to be presented in ‘gist‐based’ manner, that is, a simple format that captures the bottom‐line meaning of the message.^15^ There is relatively little research that directly examines public responses to verbatim information within materials sent out by screening programmes. Further investigation in this area is needed if people are to be adequately and equally informed about colorectal cancer screening.

### Aims of the study

We aimed to investigate how people interpret the NHS BCSP information booklet ‘Bowel Cancer Screening: The Facts’ using the ‘think‐aloud’ method. This study is part of a broader programme of NIHR‐funded research known as ASCEND. The current study will provide the basis for the design of a supplementary ‘gist‐based’ information leaflet. We therefore had a specific objective to identify areas of the existing booklet that were difficult to read, confusing to the reader or detrimental to motivation and quantify them within a typology of utterances. Our secondary objective was to identify additional responses to the information using a more in‐depth qualitative analysis.

## Methods

### Participant recruitment

Following ethical approval (ref: 2247/002), 21 participants were recruited via mail from two community organizations. Social Action for Health (SAfH) is a non‐governmental organization working within deprived communities in London. ContinYou is an education charity, working with children and adults from deprived communities across the UK. Individuals that had previously agreed to take part in studies with our research group were also recruited. Individuals were purposively sampled from deprived groups because of the established link with literacy^18^, ^19^ and colorectal cancer screening uptake.^5^

Eligible participants were sent an information sheet, consent form and freepost return envelope. Inclusion criteria were being aged 45–60 years (i.e. before the age at which colorectal cancer screening is offered in England) and no previous diagnosis of colorectal cancer. The exclusion criteria were not being able to speak or read English, previous participation in the NHS BCSP and severe cognitive impairment. These criteria were chosen to ensure that individuals were relatively naïve to the processes of colorectal cancer screening and the accompanying information materials. Three participants completed interviews, but were subsequently excluded because one was illiterate and two had cognitive impairments. Participants were paid £20 for their time and travel expenses. Interviews took place in the community or in university meeting rooms.

### The think‐aloud method

The ‘think‐aloud’ method was used within this study. It entails the verbalisation of a person's thoughts that would normally be silent, while enabling the individual to continue with the primary task (such as completing a puzzle, calculating a mathematical sum or reading textual information). The verbalized thoughts represent the current contents of short‐term memory, providing access to cognitive processes that occur during a task.^20^ A recent meta‐analysis of 94 studies and 3 500 participants has demonstrated that the method is empirically and conceptually distinct to introspection.^21^, ^22^

### Procedure

This study used a ‘marked protocol’ in which participants were prompted to make a comment every time they encountered a small red dot in the leaflet. There were a total of 66 prompts that were placed by a researcher (SS) at the end of bullet points and short paragraphs (i.e. two short sentences). Where lengthy paragraphs were included (i.e. 2–3 longer sentences), a prompt was placed after each sentence in the paragraph. A marked protocol was used as it has previously elicited more instances of confusion and miscomprehension, a primary aim of the study.^23^

**Figure 1:**
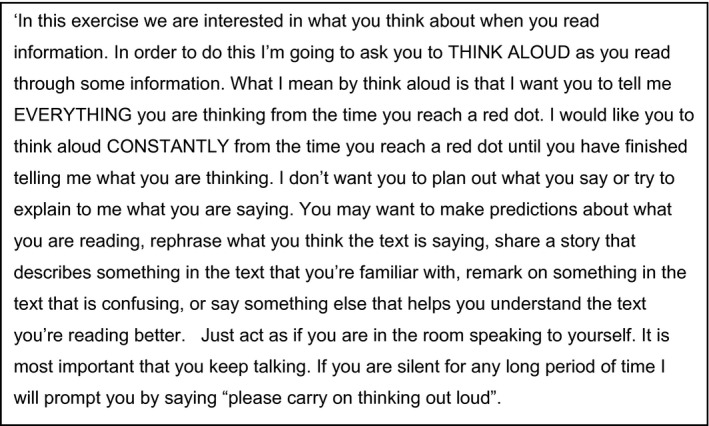
Participant instructions.

Participants were asked to complete a brief socio‐demographic questionnaire on arrival followed by the structured interview. In line with best practice for reporting think‐aloud studies,^21^ the statement in Fig. 1 (adapted from^20^, ^23^) was read to participants prior to beginning. Participants were asked to practice thinking aloud on a control leaflet (‘recycle to save the environment’), which contained three prompts before reading ‘Bowel Cancer Screening: The Facts’ (October, 2010 version).^8^ After participants had completed three successful utterances, they were deemed ready to participate. If they did not reach this threshold during the practice session, the procedure was explained again and they were given additional time to practice.

### Sample size

When determining the sample size for think‐aloud studies, it has been argued that a single test subject yields up to a third of usability problems, while after as little as five participants, most issues are identified.^24^ We therefore recruited a sample of 15–20 participants to ensure the aims of our study were met. Saturation (i.e. when no new themes or information was gained after several consecutive interviews) was used as the marker at which recruitment ceased.

### Measures of participant characteristics

Participant characteristics were recorded. These included age, gender, marital status, first language, living arrangement, employment, education‐level screening history (women only) and experience with cancer.

### Data analysis

Interviews were audio‐recorded and transcribed. Occasions when participants deviated from the text (i.e. failed to read the text or misspoke) were coded as reading mistakes. After this, prompted and unprompted utterances (any statements made following a passage of text) were coded. Participants were not instructed to make unprompted utterances prior to starting the interview. However, there was author consensus that unprompted utterances, when made, were not substantially different from prompted utterances. All utterances were therefore collapsed and analysed together. All analyses were performed in NVivo 9.

This study used a mixed‐methods approach to analyse the data. Firstly, a coding framework was developed in consultation with previous literature^23^ and the research team (SS, GV, RR, CVW, JW) (see Table 1). A content analysis was then performed, with utterances allocated to at least one theme. An utterance could be coded into several themes if deemed necessary; however where possible, multiple coding was kept to a minimum. An utterance could also be split into several sections if the participant was discussing several aspects of the text. Two of the transcripts (> 10% of the data) were second‐coded by an additional researcher (GV) to assess inter‐rater reliability. Reliability was found to be adequate to excellent (*K *=* *0.5–1.0).

**Table 1:** Coding framework

Name of theme	Description
Deep processing	An inference based on the text, which goes beyond repetition Rephrasing of the text, which goes beyond repetition An anecdote which explains the text
Surface processing	Repetition or very near repetition of the text Self‐reported learning Self‐reported previous knowledge
Miscomprehension	Confusion about a statement An incorrect statement following a passage of text Asserts that factual information is opinion
Emotional (negative)	A negative reaction with at least one emotion in the sentence Person mentions the information makes them feel the opposite of a positive emotion
Emotional (positive)	A positive reaction with at least one emotion in the sentence Person mentions the information makes them feel the opposite of a negative emotion
Unanswered questions	An individual has unanswered questions following a passage
Layout	An individual comments on the layout of the information
Unnecessary information	Comments that indicate the information is unnecessary
Decrease motivation	An individual remarks that something in the text would be demotivating to screening participation
Increase motivation	An individual remarks that something in the text would be motivating to screening participation

In addition to the content analysis, an in‐depth thematic analysis was conducted to provide insight into the subthemes contained within the framework. Thematic analysis is used to identify, analyse and report patterns (themes) within data.^25^ While the majority of the comments were brief, and provided little insight past surface‐level meaning, a thematic analysis allowed exploration of deeper‐level meanings of some comments. This approach was taken as the aims of the study were to extract general perceptions about The Facts booklet, rather than understand individual experience with the information.

To increase the validity of the thematic analysis, two researchers were responsible for analysing the transcripts (SS and CvW). SS analysed each individual interview and extracted themes. Themes within each interview were categorized by two researchers (SS and CvW) and analysed across transcripts using the constant comparison method.^26^ To increase the validity further, the wider study group were responsible for suggesting alternative themes within the data and to assess whether the suggested themes were adequately represented by the quotes.

## Results

### Participant characteristics

A total of 18 participants [mean age = 55 years (range 48–60)] took part. As indicated by Table 2, the sample was mixed; participants predominantly spoke English as a first language, were of white ethnicity, had a mixed level of education and most had experience of cancer in some form.

**Table 2:** Participant characteristics

Participant characteristic	*n* (%)
Gender	
Male	7 (39)
Female	11 (61)
Marital Status	
Married/living with partner	6 (33)
Single/divorced/separated	9 (50)
Widowed	3 (17)
English as first language	18 (100)
Living arrangement	
Own home/mortgage	9 (50)
Renting/Other	9 (50)
Employment	
Currently employed	10 (56)
Unemployed/disabled or too ill to work	6 (33)
Retired	2 (11)
Education	
≤ GCSE or O‐Level	4 (22)
> GCSE or O‐Level	14 (78)
Ethnicity^a^	
White	15 (83)
Non‐white	2 (11)
Previous cancer diagnosis	
Yes	2 (11)
No	16 (89)
Know at least one person diagnosed with cancer	
Yes	15 (83)
No	3 (17)
Breast screening history^b^	
Yes	11 (100)
No	0 (0)
Cervical screening history^b^	
Yes	10 (91)
No	1 (9)

a One participant elected not to answer this item.

b Women only.

### Content analysis

In the 18 interviews, 270 reading mistakes were recorded (mean = 15 per person; range = 0–59). The interviews yielded 776 coded utterances (mean = 43.1 per person; range = 8–95), which were analysed within the pre‐determined framework.

**Figure 2:**
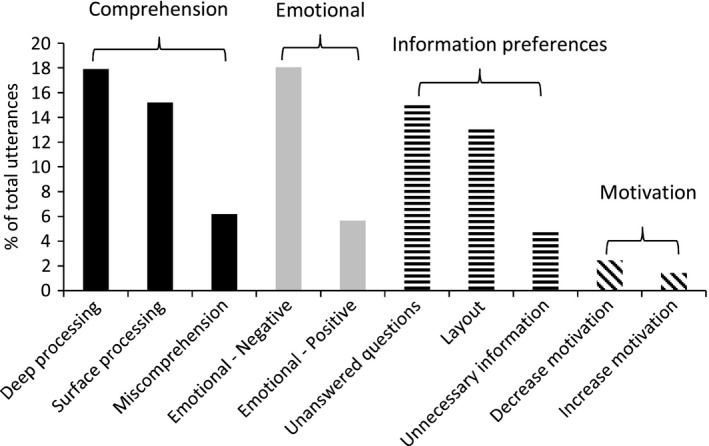
Typology of participant utterances.

There was substantial variation in the types of comments made by participants (see Fig. 2). The comprehension theme was largely made up of comments which implied higher‐level understanding (i.e. deep processing; 17.9% of all comments), or repetitions of the text and unsubstantiated self‐reported knowledge (i.e. surface processing; 15.2%). Miscomprehension was less common (6.2%); however, this still amounted to 48 instances of mistakes or self‐reported lack of understanding. There were a high number of comments in the emotional theme. Emotionally negative statements were three times more common than emotionally positive statements (18.0% and 5.7%, respectively). The information preferences theme suggested that people desired further information on specific aspects of the booklet (unanswered questions: 15.2%), while others suggested improvements to the style and layout of the booklet (layout: 13.1%). A minority of statements questioned the necessity of certain information that they had just read (unnecessary information: 4.8%). Utterances rarely alluded to whether the participant felt motivated (1.4%) or demotivated (2.5%) by information in the booklet.

### Thematic analysis

#### Difficulties with numerical information

The use of numbers to convey risk information in ‘The Facts’ booklet is common, which participants often considered to be unnecessary. For example, one participant preferred to think categorically about the efficacy of screening to reduce colorectal cancer deaths (i.e. anything is better than nothing), rather than in verbatim terms (i.e. a 16% reduction): *‘I know we have to have…the evidence and that, but I think if I hadn't done research myself…I would just find that got in the way really. This thing about 16%. What's 16%? What does it mean to the person on the street? I know anything is better than nothing for reducing the risk of dying, but surely it should be a lot more percentage than that, but is it something that I want to know about?'* (QE, 50 years, female, degree‐level education).

The use of numerical information to convey the lifetime incidence of colorectal cancer as 1 in 20 led to confusion. For example, one participant largely overestimated the likelihood of being diagnosed with colorectal cancer as a result of an information‐processing error: *‘That's about, yea, that's one in four of the population isn't it?’* (IT, 51 years, male, higher educational qualifications).

The prevalence of screening outcomes proved difficult to interpret. The booklet explains that following an FOB test, approximately 98 of 100 individuals will receive a normal result (no blood found), four of 100 will receive an unclear result (a small amount of blood), and two of 100 receive an abnormal result (blood was found, further investigation is required). However, there was confusion as to whether the normal prevalence figure includes those that have previously received an unclear result: *‘Does that equate with the 98 out of 100 in the previous paragraph? Something, somewhere doesn't seem quite. Four people out of 100 and then we had 98 out of 100, anyway, not quite sure about that’* (WW, 56 years, female, degree‐level education).

As with the FOB results section, colonoscopy outcomes were misinterpreted. The booklet explains that one person of 10 will be diagnosed with cancer, four people of 10 will have a polyp removed and five people of 10 will have nothing found. In this instance, the participant appears to discount the number of people receiving a polyp diagnosis, thus overestimating the prevalence of cancer following an abnormal FOB result: *‘Half of people that go for these colonoscopes (sic) don't have cancer? And the other half do? Hmm’* (IT, 51 years, male, higher educational qualifications).

#### Unfamiliar topics and complex terminology

Participants questioned whether it was necessary to have such a long and complex booklet to inform people about the screening programme: *‘This is an awful lot for people to read, this is just handed out? Hell of a lot to read isn't it?’* (OU, 54 years, female, degree‐level education). Comments were often made due to the introduction of unfamiliar topics and scientific terminology, ‘*A bit difficult to understand, if you're not up to date with those kind of informations*’ (RT, 58 years, female, no formal qualifications). This often led to difficulties with pronunciation (see Table 3 for a list of the most common reading mistakes). Participants argued that a leaflet which aimed to provide complex and technical information would benefit from the use of vernacular language as opposed to scientific terminology: *‘… I would prefer a more high level definition of what the bowel is actually. This just seems to provide too much detail…’* (SM, 51 years, male, degree‐level education).

**Table 3:** Prevalence of reading mistakes

Word or phrase	*n*
Polyp	7
Faecal	9
Adenoma	10
Colorectal	10
Colonoscope	11
Colonoscopy	49

There was also difficulty when describing the difference between the possible outcomes of an FOB test. Despite the bold text within this paragraph describing the exact meaning of abnormal, it was easily misinterpreted as the definite identification of a malignancy or polyp: *‘So that's good, it gives you all of the different results of the testing…normal, you're not going to have any more tests for 2 years. If it's unclear you have another one to make sure it's nothing suspicious and if it's abnormal you've definitely got something that needs further investigation.’* (CW, 56 years, female, degree‐level education).

This and other complex areas of the booklet were improved by the provision of summary boxes and diagrams. To improve the booklet further, it was recommend that when technical phrases are introduced, the most familiar word should be used first, and the more technical phrase included within brackets that follow: *‘I'm wondering sometimes with these things whether it isn't better to have the common word before the technical, so piles (haemorrhoids), just because seeing those words that are hard to pronounce can put you off.’* (OU, 54 years, female, degree‐level education).

#### Emotional responses

As demonstrated by the quantitative analysis, there was a mixture of emotionally negative and positive comments. For example, some participants found the scientific explanations of cancer interesting, and somewhat reassuring: *‘yea that's interesting, I've never really known an awful lot about cancer, and how it spreads and what happens so that again seems to make it quite sensible and slightly not too scary. Because obviously everybody talking about cancer, everybody gets very “the big C”’* (WW, 56 years, female, degree‐level education).

Despite the reassurance offered by these explanations, the colonoscopy risk information frequently led to negative emotional responses. In particular, the risk of death (1 in 10 000) led some to question why this may occur: *‘Oh, oh that is shocking. That is shocking. I'd like to know more, now that's been said…what on earth would they have had to do for that to happen – whether a heart attack, or a shock to the body or you perforate the liver or something that's vital to keep you alive’* (CW, 56 years, female, degree‐level education). Others questioned the necessity of including such information, preferring instead to supply it on a ‘need to know’ basis or in a less prominent position: *‘I'd write it in small and I'd write it at the end…It wouldn't be something massive, I don't think it, anything put there to make people more worried about the procedure, the procedure's complicated enough’* (JS, 52 years, male, A‐Level education)

The nature of the test was often considered to be distasteful: *‘yea I think that probably, there's nothing else you can do about it but it is rather embarrassing and unpleasant’* (BD, 56 years, female, no formal qualifications). One individual commented that the description evoked unpleasant images about the procedure that may induce aversion to participation: *‘ok, yea, wipe the samples on a special card…I'm getting a bit unpleasant mental images of that procedure’* (SM, 51 years, male, degree‐level education).

## Discussion

This study of 18 adults who were naïve to colorectal cancer screening explored how people interpret the information booklet provided to invitees of the English NHS BCSP. Despite the extensive testing process the information went through^14^ and its approval by the plain English campaign, our mixed‐methods analysis suggests it may not always meet the information needs of some older adults. Furthermore, this gap in understanding is not filled by health‐care professional counselling, suggesting that communication inequalities may be created through the introduction of home‐based organized screening programmes.^27^

Participants made on average 15 reading mistakes during the task, and in line with previous research in Australia, unfamiliar words such as colonoscopy, colorectal and adenoma were particularly problematic.^28^ The introduction to the function of the colon and rectum and the adenocarcinoma sequence necessitated the use of such terminology, leading some participants to question whether it should be included. Importantly, these basic scientific explanations stretched the capabilities of even highly educated participants.

Our observations concur with medical decision‐making theory,^15^ which recommends that where possible, the ‘gist’ of information is presented as opposed to literal facts. In keeping with recommendations from the risk communication literature,^29^ participants preferred numerical information to be presented in the most simple format (i.e. high vs. low), rather than precise risk information (i.e. 16% relative risk reduction). Yet even in cases where recommended numerical presentation styles for probabilities were present (i.e. 1 in 20),^30^, ^31^ some participants reported and demonstrated confusion. While there is a tendency to believe that the provision of more information will improve knowledge,^17^ emerging evidence suggests ‘less is more’ when it comes to health information and medical decision making.^15^, ^16^, ^32^, ^33^, ^34^, ^35^, ^36^ Our qualitative data support this. For example, it was suggested that information about the ability of the test to detect polyps was of less importance to a booklet primarily about colorectal cancer. Participants also recommended that numerical information about unclear results and colonoscopy risk was reduced or simplified.

It was surprising that the booklet elicited frequent emotional responses, the majority of which were negatively framed such as fear of the possible outcomes and worry following risk information. As with previous qualitative research,^14^ risk information relating to colonoscopy was considered to be particularly shocking and in some cases unwanted. However, in Woodrow and colleagues' study, only a minority of individuals were found the hold such views. The quantitative element of the current study demonstrates clearly that such views may be more prevalent than previously thought.

In line with previous questionnaire‐based research, the disgust and ‘messiness’ of the FOB testing procedure was a common reaction and could potentially act as a barrier to screening for some individuals.^11^, ^37^, ^38^ Despite the higher prevalence of negative emotional responses, very few utterances suggested that this information was demotivating. This finding is welcomed in the light of the booklet's aim to improve informed choice in screening decisions.

This was the first study to use the think‐aloud method to evaluate the quality of written information in the largest organized cancer screening programme worldwide. Our mixed‐methods approach enabled us to present a broad overview of public perceptions of the information materials, as well a more detailed analysis of the underlying factors which may contribute to decision making in screening. The inclusion of individuals that spoke English as a first language allowed us to focus on literacy and not translation, which are considered to be separate issues.^28^ Furthermore, participants were approaching screening age, but had not yet been screened thus preventing biases that may occur in individuals with more experience of the screening procedure and information materials. However, it is also possible that individuals reading the information in a hypothetical context for a distant future behaviour may have viewed the materials differently to someone making a current decision about screening.^39^

This study has additional limitations that should be considered. While an objective of the study was to identify difficulties that individuals have with reading and evaluating the study materials, it was people with low literacy skills who were particularly challenged by this method. Due to the stigma associated with poor basic skills,^40^, ^41^ a number of interviewees found the approach quite intimidating and stressful. Although these individuals were thoroughly debriefed following the interview, best practice guidelines for think‐aloud research do not allow researcher involvement until this point.^20^ Furthermore, as reflected in the quotes outlined above, the more educated participants produced the most revealing utterances and contributed disproportionately to the discussion. While the method was useful at highlighting areas of the booklet that were difficult to interpret to the general population, the think‐aloud method may need to be adapted or other strategies employed when attempting to identify the specific needs of individuals with low literacy.

The use of a marked protocol may have encouraged utterances related to miscomprehension.^23^ However, it may also have introduced bias by encouraging comments at points in the booklet dictated by the researchers and discouraging them at others. Further research comparing marked and unmarked protocols is needed to clarify these issues.

The sample reported here was also relatively experience with cancer and cancer screening. For example, a nationally representative sample of UK older adults reported that 74% of individuals knew someone with cancer or had been diagnosed themselves, compared to 89% in the current sample.^42^ There were also a high percentage of women who had previously participated in both breast (100%) and cervical (91%) cancer screening programmes. This familiarity with cancer and cancer screening may have accounted for the relative lack of negative statements and could limit the degree to which our findings are generalisable outside of this study population.

This study has implications for the NHS BCSP, as well as for other researchers investigating public perceptions of health services. For example, the findings reported here will be incorporated into a wider NIHR‐funded project that aims to design a supplementary ‘gist‐based’ information leaflet.^43^ This leaflet is designed to be an easy to read source of information about the NHS BCSP and will be presented in a format that matches preferred processing styles.^15^ The leaflet will be evaluated in a national randomised controlled trialled within the existing NHS BCSP.

In addition, we were able to demonstrate that the think‐aloud method is a simple, yet effective strategy for evaluating health information materials among educated individuals. Considering that small samples can still elicit a large proportion of usability issues,^24^ researchers testing communication materials (e.g. information sheets, consent forms, questionnaires or multimedia resources) could easily implement this technique within the recommended stages of information design.^44^ However, care must be taken when using the technique with low literacy individuals and future research should investigate alternative, less stressful tasks for such groups.

## Conclusion

The think‐aloud method enabled us to successfully identify specific areas of the existing information materials that were difficult to read, confusing to the reader and detrimental to motivation. We also observed strong emotional responses to some aspects of the screening process. Supplementary information that takes our findings into consideration may aid knowledge translation and reduce the cognitive burden placed on individuals when deciding whether or not to take up the offer of colorectal cancer screening.

## Source of funding

This paper summarizes independent research funded by the National Institute for Health Research (NIHR) under its Programme Grants for Applied Research Programme (Grant Reference Number (RP‐PG‐0609‐10106). The views expressed in this article are those of the authors and not necessarily those of the NHS, the NIHR or the Department of Health. Mr Smith is supported by a PhD studentship from the Medical Research Council (United Kingdom).

## Conflict of interest

No conflicts of interest have been declared.
